# The Incidence and Survival Rate of Population-Based Pancreatic Cancer Patients: Shanghai Cancer Registry 2004–2009

**DOI:** 10.1371/journal.pone.0076052

**Published:** 2013-10-09

**Authors:** Jianfeng Luo, Linhai Xiao, Chunxiao Wu, Ying Zheng, Naiqing Zhao

**Affiliations:** 1 Department of Health Statistics and Social Medicine, School of Public Health, Fudan University, Shanghai, China; 2 Department of Cancer Control & Prevention, Shanghai Municipal Center for Disease Prevention & Control, Shanghai, China; The University of Hong Kong, China

## Abstract

**Background:**

Pancreatic cancer is a devastating disease with dismal prognosis. Large population-based evidence on its survival rate and influence factors is lacking in China.

**Objective:**

This study aimed to depict the demographic factors, tumor characteristics, incidence rate and survival rate of pancreatic cancer cases in urban China.

**Methods:**

The demographic factors, tumor characteristics were collected for all the pancreatic cancer cases identified during 2004 to 2009 from the Shanghai Cancer Registry. The survival time was ascertained through linkage of the Shanghai Cancer Registry and the Shanghai Vital Statistics Registry. The deadline of death certificates was the end of December 2012. Kaplan-Meier method and Cox proportional-hazards regression model were used to explore the survival rate and influence factors.

**Results:**

11,672 new pancreatic cancer cases were identified among Shanghai residency during 2004 to 2009. The crude incidence rate of pancreatic cancer was increasing from 12.80/100,000 in 2004 to 15.66/100,000 in 2009, while the standardized incidence rate was about 6.70/100,000 and didn't change a lot. The overall 5-year survival rate was 4.1% and the median survival time was 3.9 (95% Confidence Interval (CI) 3.8–4.0) months. Subjects had received surgical resection had improved survival (HR  = 0.742, 95% CI: 0.634–0.868) than its counterparts. In adjusted multivariable Cox proportional-hazard models, factors associated with poor survival included older age at diagnosis (age > = 70 years: hazard ratio (HR)  = 1.827, 95% CI: 1.614–2.067), male sex (HR  = 1.155, 95% CI: 1.041–1.281), distant disease at diagnosis (HR =  1.257, 95% CI: 1.061–1.488), positive lymph node (HR  = 1.236, 95% CI: 1.085–1.408), tumor stage (Stage IV HR  = 2.817, 95% CI: 2.029–3.909).

**Conclusion:**

The age-adjusted incidence rate was stable and overall survival rate was low among pancreatic cancer patients of Shanghai residency. Early detection and improved treatment strategies are needed to improve prognosis for this deadly disease.

## Introduction

Pancreatic cancer (PC) is a devastating disease and the fourth, fifth and seventh leading cause of cancer-related death in the United States, European Union and China, respectively [1], [2], [3]. It has a dismal prognosis. Large population-based studies in western countries indicated an overall 5-year survival rate less than 5% and a median survival of 3–6 months [4], [5], [6], [7]. The age-adjusted pancreatic adenocarcinoma incidence of 18 or older was about 11 per 100,000, the median survival rate was 4 months and the 2-year survival rate increased from 4.6% in 1988 to 7.7% in 2000 according to the Surveillance, Epidemiology, and End Results registry(SEER) from 1988 through 2002 [4]. Another study using SEER registry indicated that the incidence rate for pancreatic head cancer was 5.6 per 100,000, whereas the rate for pancreatic body/tail cancers was 1.6 per 100,000 between 1973 and 2002. The 3-year survival rate for pancreatic body/tail cancer is 3.4% compared with 5.3% for pancreatic head cancer [6]. The National Cancer Database from 1989 through 1995 indicated that the overall 5-year survival rate for pancreatic cancer was 23.4% for patients who had pancreatectomy, compared with 5.2% for those who had no cancer-directed treatment [8]. The median survival time for pancreatic cancer cases reported during 1996 to 2000 from the population-based Alabama Statewide Cancer Registry was 0.39 years [7]. One-year and 5-year survival rates for pancreatic adenocarcinoma were 16% and 2% respectively, while the median overall survival was 4 months for pancreatic adenocarcinoma in the population-based California Cancer Registry [5].

With the improvement of skill of surgeons and wide application of vascular surgery, the resection rate of pancreatic cancer has been increased to 20% in China [9]. The survival rate of pancreatic cancer increased in the past 20 years and was comparable to the western countries according to several recent hospital-based studies [10], [11], [12], [13]. A prognostic study in Chinese patients with pancreatic cancer indicated that the median survival rates were 17.2 months and 9.7 months for the EGF (-) EGFR (-) group and EGF (+) EGFR (+) group, respectively [13]. The 5-year overall survival rate for pancreatic cancer who underwent pancreaticoduodenectomy increased from 8% to 19% over 10 years period from 2000 to 2009 in Tianjin Medical University Cancer Institute and Hospital [10]. Overall median survival time was 4.7 months and 1-year overall survival rate was 14% for pancreatic cancer with liver metastases [11]. The 3-year and 5-year overall survival rate were 6% and 2% respectively and the median survival time was about 10 months for pancreatic ductal adenocarcinoma in a case control study among the Han Chinese ethnic group [12].

A retrospective survey on 2340 pancreatic cancer subjects from 14 large-scale hospitals in 8 provinces and 2 metropolises in China indicated that the median survival time of radical resection of carcinoma in the head of pancreas was 17.11 months and the 5-year survival rates was 8.47% [9]. However, results from these hospital-based studies, including estimates of survival, are unlikely to be generalizable to the entire population of pancreatic cancer patients. Population-based studies that include all cases would provide more accurate estimates of survival for the general population of pancreatic cancer patients. To our knowledge, there are no population-based studies in Chinese population focused on estimating overall survival rates of pancreatic cancer and its demographic and prognostic clinical factors.

Thus, we performed a population-based study using the Shanghai Cancer Registry to evaluate the survival of patients with pancreatic cancer. The aims of this investigation were as follows: (1) to describe the demographic, tumor, and treatment characteristics of patients diagnosed with pancreatic cancer; (2) to analyze prognostic factors influencing pancreatic cancer survival.

## Subjects and Methods

### Ethics statement

This study was approved by the Ethics Committee of the School of Public Health, Fudan University, Shanghai, China. No written consent form was obtained since we used the registry data. No written consent was given by the patients for their information to be stored in the database and used for research since it was specifically waived by the approving IRB.

### The Shanghai Cancer Registry and the Shanghai Vital Statistics Registry

The Shanghai Cancer Registry, a population-based cancer registry, was initiated and established to collect and analyze data of cancer incidence, mortality and survival of residency in Shanghai since 1963. According to the regulation issued by the Shanghai Municipal Bureau of Public Health, all medical facilities (more than 150 units) in Shanghai are responsible for notifying all newly diagnosed cancer cases as well as cases of benign tumors of the central nervous system to the registry. A standardized notification card, which includes information on name, date of birth, sex, address, occupation, cancer site, date and basis of cancer diagnosis, is used for reporting cancer cases. The notifications completed by physicians or medical clerks are sent to the cancer registry and then placed on file according to the name of the patient and administrative district of residence. Home visits are carried out every month to confirm if the cancer patient is a permanent resident of the registration coverage area [14]. Follow-up visits are also conducted by the center of community health service for treatment and survival information.

Death certificates for all cancer patients are obtained bilaterally from follow-up system and from the Vital Statistics Section of the Shanghai Municipal Center of Disease Control and Prevention and collated with the file of new cases kept in the registry. If the deceased was not registered prior to death, the registry staff would interview the relatives of the case to obtain information on the hospital where the case was diagnosed and treated, the date and basis of cancer diagnosis. Such information is also collected from the hospital if there is any doubt about the accuracy of information provided by the relatives. Since late 1980s, the registry has obtained information on vital status of cancer patients by active and passive follow-up [14].

### Cases Identification and Survival Time of Pancreatic Cancer

From the Shanghai Cancer Registry, we identified patients from 2004 to 2009 diagnosed with pancreatic cancer. Patients with pancreas listed as the primary disease site were identified using the ICD-10 codes C25.0–25.9. Data on patient demographics, year of diagnosis, site, TNM stage, historical status, tumor grade were extracted. The survival time was ascertained through linkage of the Shanghai Cancer Registry and the Shanghai Vital Statistics Registry. The deadline of death certificates was the end of December 2012, so all patients were followed for at least 4 years.

### Statistical analysis

The incidence rates of pancreatic cancer from 2004 to 2009 were calculated by the new pancreatic cancer cases identified in the Shanghai Cancer Registry and the number of Shanghai residency extracted from the Shanghai Statistical Yearbook. The standardized incidence rates were calculated using age-specific incidence rate and the international age structure [15].

Kaplan-Meier survival estimates and log rank test were used to depict and compare the survival rates of pancreatic cancer by age at diagnosis, sex, cancer stage, grade, tumor site, and treatment (surgical resection vs. non-surgical resection). Since there is no information about treatment on cancer in the Shanghai Cancer Registry, those subjects with pathology data were regarded as ‘surgical resection’ while the others were regarded as ‘non-surgical resection’. Cox proportional-hazards regression models were applied to estimate the hazard ratios and 95% confidence intervals for the association between surgical resection and survival of pancreatic cancer, with adjustment for demographic factors and tumor characteristics. Statistical tests were 2 sided and considered statistically significant for P<0.05. Statistical analyses were conducted using SAS version 9.3 (SAS Institute, Inc., Cary, North Carolina).

## Results

### The incidence rate of pancreatic cancer

**Figure 1:**
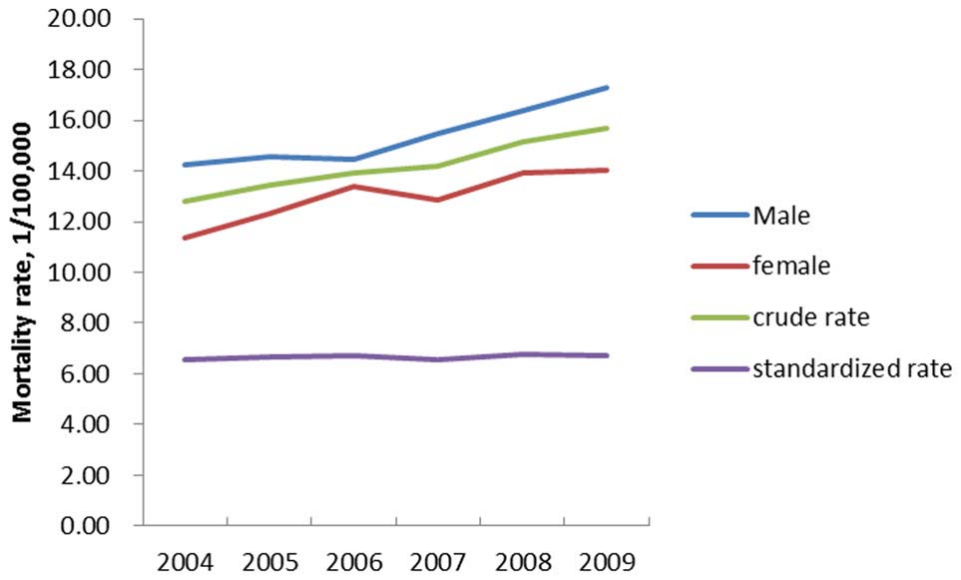
The incidence rate of pancreatic cancer of Shanghai residency during 2004–2009 (1/100,000).

There were 11672 new identified pancreatic cancer cases in the Shanghai Cancer Registry during 2004–2009. The crude incidence rate of pancreatic cancer increased from 12.80/100,000 to 15.66/100,000 in 2004 to 2009. However, the standardized incidence rate didn't change a lot in the same period (Table 1 and Figure 1). Male had higher incidence rate of pancreatic cancer than female, with the incidence rate 17.28/100,000 and 14.04/100,000 respectively, in 2009.

**Table 1:** The incidence rate of pancreatic cancer of Shanghai residency during 2004–2009 (1/100,000).

	2004	2005	2006	2007	2008	2009
crude rate	12.80	13.46	13.93	14.19	15.15	15.66
Male	14.23	14.58	14.45	15.49	16.40	17.28
female	11.35	12.32	13.41	12.88	13.90	14.04
standardized rate*	6.55	6.68	6.72	6.58	6.78	6.71

* Standardized by international age structure.

### Demographic, tumor characteristics

The distributions of patient demographic, tumor characteristics are shown in Table 2. The median age at diagnosis was 72 years, with 20.7% of patients less than 60 and nearly 60% of patients over 70 years old. Approximately 54.4% of patients were men. Tumor location was available for 36.7% of patients, with the majority of tumors located in the head (30.7%), followed by tail (3.4%) or body (2.6%), of the pancreas. Nearly 42.9% had regional or distant metastasis disease at diagnosis, whereas only 49.2% had localized disease. Tumor grade (available for 676 out of 11672) indicated advanced disease at diagnosis, with approximately 57.5% of tumors moderately differentiated, 33.7% poorly differentiated, 1.2% undifferentiated and only 7.5% characterized as well differentiated. Tumor stage also indicated advanced disease at diagnosis, with 66.2% stage IV, 18.2% stage III, 11.5% stage II and 4.0% stage I.

**Table 2:** Demographic factors, tumor characteristics of newly identified pancreatic cancer cases of Shanghai residency during 2004–2009.

		All subjects (n = 11672)	Surgical resection (n = 676)	Without surgical resection (n = 10996)	p value
sex	Male	6351 (54.4%)	397 (58.7%)	5954 (54.1%)	0.0203
	Female	5321 (45.6%)	279 (41.3%)	5042 (45.9%)	
Age of diagnosis	N	11672 (0)	676 (0)	10996 (0)	
	Mean±SD	70.1±12.0	61.6±10.5	70.6±11.9	<0.001
	Median	72	62	73	
	P25–P75	62.0∼79.0	55.0∼70.0	63.0∼79.0	
	Min – Max	5.0∼101.0	18.0∼87.0	5.0∼101.0	
Age groups of diagnosis	<60	2415 (20.7%)	293 (43.3%)	2122 (19.3%)	<0.001
	60–70	2353 (20.2%)	202 (29.9%)	2151 (19.6%)	
	70–	6904 (59.2%)	181 (26.8%)	6723 (61.1%)	
Tumor site	Head	3578 (30.7%)	337 (49.9%)	3241 (29.5%)	<0.001
	Body	306 (2.6%)	45 (6.7%)	261 (2.4%)	
	Tail	397 (3.4%)	31 (4.6%)	366 (3.3%)	
	Not specified/unknown	7391 (63.3%)	263 (38.9%)	7128 (64.8%)	
Tumor grade	undifferentiated	8 (1.2%)	8 (1.2%)		
	poorly differentiated	228 (33.7%)	228 (33.7%)		
	moderately differentiated	389 (57.5%)	389 (57.5%)		
	well differentiated	51 (7.5%)	51 (7.5%)		
TNM-Tumor	0	46 (0.4%)	2 (0.3%)	44 (0.4%)	
	1	262 (2.2%)	59 (8.7%)	203 (1.8%)	<0.001
	2	492 (4.2%)	114 (16.9%)	378 (3.4%)	
	3	643 (5.5%)	101 (14.9%)	542 (4.9%)	
	4	609 (5.2%)	66 (9.8%)	543 (4.9%)	
	X	443 (3.8%)	16 (2.4%)	427 (3.9%)	
	Not specified/unknown	9172 (78.6%)	317 (46.9%)	8855 (80.5%)	
	is	5 (0.0%)	1 (0.1%)	4 (0.0%)	
TNM-Node	0	879 (40.8%)	167 (56.0%)	712 (38.3%)	
	1	721 (33.4%)	114 (38.3%)	607 (32.7%)	<0.001
	X	556 (25.8%)	17 (5.7%)	539 (29.0%)	
TNM-metastasis	0	1248 (49.2%)	266 (76.4%)	982 (44.8%)	
	1	1089 (42.9%)	76 (21.8%)	1013 (46.2%)	<0.001
	X	202 (8.0%)	6 (1.7%)	196 (8.9%)	
Stage	0-I	153 (4.0%)	41 (9.8%)	112 (3.3%)	<0.001
	II	434 (11.5%)	129 (30.9%)	305 (9.1%)	
	III	690 (18.2%)	127 (30.4%)	563 (16.7%)	
	IV	2505 (66.2%)	121 (28.9%)	2384 (70.9%)	

The subjects underwent surgical resection had younger age (median: 62 vs. 73, p<0.001), more carcinoma in the head of pancreas (49.9% vs. 29.5%, p<0.001), higher TNM score on tumor size, more negative lymph node (56.0% vs. 38.3%, p<0.001), less metastasis (21.8% vs. 46.2%, p<0.001) and lower stage (p<0.001) than their counterparts.

### Survival rate and prognostic factors

**Figure 2:**
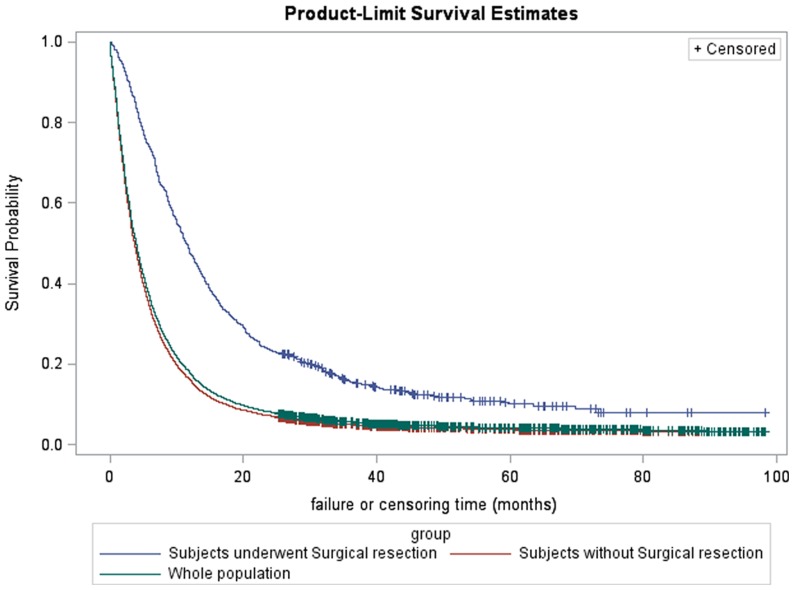
The survival rate of pancreatic cancer patients among Shanghai residency, Shanghai Cancer Registry 2004–2009. Whole population: 1-year survival rate 17.8%, 3-year survival rate 5.7%, 5-year survival rate 4.1%, Subjects underwent surgical resection: 1-year survival rate 47.5%, 3-year survival rate 15.4%, 5-year survival rate 10.1%, Subjects without surgical resection: 1-year survival rate 16%, 3-year survival rate 5.1%, 5-year survival rate 3.7%.

**Figure 3:**
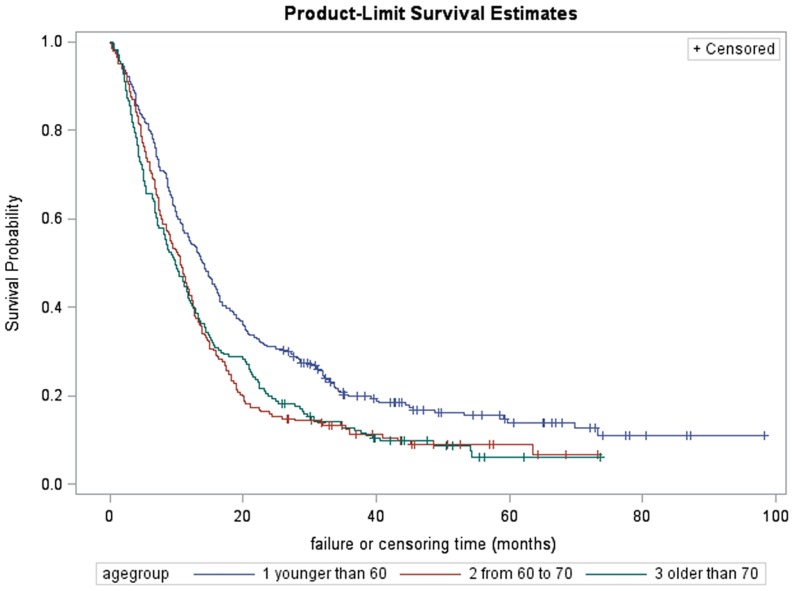
the survival rate of pancreatic cancer patients among Shanghai residency underwent surgical resection, Shanghai Cancer Registry 2004–2009. Age <60: 1-year survival rate 54.8%, 3-year survival rate 19.8%, 5-year survival rate 13.9%, Age <70: 1-year survival rate 42.3%, 3-year survival rate 11.2%, 5-year survival rate 6.7%, Age>70: 1-year survival rate 40.8%, 3-year survival rate 12.3%, 5-year survival rate 6.2%.

The median survival time was shown in table 3 and plots of the product-limit estimates for survival were depicted for the whole sample and by surgical resection (Figure 2). The survival rate of those subjects underwent surgical resection was shown by age groups in Figure 3. The median survival time for all the subjects was 3.9 months (95% confidence interval (CI) 3.8–4.0 months) with 1-year survival rate 17.8%, 3-year survival rate 5.7% and 5-year survival rate 4.1%. The subjects had received surgical resection had better survival than their counterparts, with median survival time 11.5 months vs. 3.6 months and 5-year survival rate 10.1% vs. 3.7% respectively. Younger patients underwent surgical resection had improved survival rate, while some subjects older than 70 received surgical resection also had good prognosis, with 5-year survival rate 6.2% (Figure 3). While male and female had no statistically significant difference in survival time, younger subjects (6.3 months (95% CI: 6.0–6.7)), well differentiated tumor (35.8 months (95% CI: 14.1–63.6)), localized disease (8.4 months (95% CI: 7.7–9.1)), node negative (8.1 months (95% CI: 7.4–8.7)), lower stage (12.2 months (95% CI: 8.9–16.7)), carcinoma in the body of pancreas (5.9 months (95% CI: 5.1–6.7)) had longer survival time than their counterparts.

**Table 3:** The median survival time and influence factors of newly diagnosed pancreatic cancer cases of Shanghai residency during 2004–2009.

	Stratum	Median (95% CI)*	P value	HR (95% CI)**	P value
Total		3.9 (3.8, 4.0)			
Surgery	Yes	11.5 (10.5,12.4)	<.0001	0.742 (0.634–0.868)	0.0002
	None	3.6 (3.5, 3.8)		ref	
Sex	Male	3.8 (3.7, 4.0)	0.4337	1.155 (1.041–1.281)	0.0064
	Female	4.0 (3.8, 4.2)		ref	
Age group	<60	6.3 (6.0, 6.7)	<.0001	ref	
	60–70	4.7 (4.5, 5.0)		1.344 (1.168–1.546)	<.0001
	70-	3.0 (2.9, 3.1)		1.827 (1.614–2.067)	<.0001
Tumor site	Head	4.7 (4.5, 5.0)	<.0001	ref	
	Body	5.9 (5.1, 6.7)		0.751 (0.557–1.012)	0.0601
	Tail	3.3 (3.0, 3.9)		0.775 (0.585–1.027)	0.0760
	Not specified/unknown	3.4 (3.3, 3.6)		0.968 (0.862–1.087)	0.5789
Histology	undifferentiated	5.2 (1.1,12.0)	<.0001		
	poorly differentiated	7.3 (6.7, 8.8)			
	moderately differentiated	13.6 (12.2,15.0)			
	well differentiated	35.8 (14.1,63.6)			
T grade	0	4.8 (2.9, 5.9)	<.0001	1.398 (0.831–2.352)	0.2071
	1	7.0 (5.5, 8.7)		ref	
	2	7.8 (6.7, 8.7)		0.846 (0.679–1.055)	0.1371
	3	6.1 (5.6, 6.8)		0.786 (0.634–0.973)	0.0273
	4	4.8 (4.2, 5.3)		0.796 (0.637–0.994)	0.0437
	X	3.9 (3.2, 4.5)		0.82 (0.651–1.034)	0.0940
	Not specified/unknown	3.5 (3.4, 3.6)		2.028 (1.084–3.795)	0.0270
	is	4.2 (1.3,27.8)		1.414 (0.521–3.837)	0.4967
N grade	0	8.1 (7.4, 8.7)	<.0001	ref	
	1	5.4 (4.8, 6.0)		1.236 (1.085–1.408)	0.0014
	X	3.9 (3.3, 4.3)			
M grade	0	8.4 (7.7, 9.1)	<.0001	ref	
	1	3.5 (3.2, 3.8)		1.257 (1.061–1.488)	0.0081
	X	4.1 (3.3, 4.9)		1.334 (1.013–1.757)	0.0401
Tumor stage	0-I	12.2 (8.9,16.7)	<.0001	ref	
	II	11.8 (10.5,13.1)		1.44 (1.066–1.945)	0.0174
	III	6.9 (6.4, 7.8)		1.909 (1.411–2.582)	<.0001
	IV	3.0 (2.9, 3.2)		2.817 (2.029–3.909)	<.0001

* median and 95% CI was estimated using Kaplan Meier curve by each variable individually.

** Hazard Ratio and 95% CI was estimated using multiple Cox regression model adjusted by surgical resection, age group, sex, site of tumor, TNM grade and stage.

Prognostic factors related to survival among the pancreatic cancer patients were also shown in Table 3. After adjusted by demographic factors and tumor characteristics, subjects had received surgical resection had improved survival (HR  = 0.742, 95% CI: 0.634–0.868) than its counterparts. In adjusted multivariable Cox proportional-hazard models, factors associated with poor survival included older age at diagnosis (age > = 70 years: hazard ratio (HR)  = 1.827, 95% CI: 1.614–2.067), male sex (HR  = 1.155, 95% CI: 1.041–1.281), distant disease at diagnosis (HR  = 1.257, 95% CI: 1.061–1.488), positive lymph node (HR  = 1.236, 95% CI: 1.085–1.408), tumor stage (Stage IV HR  = 2.817, 95% CI: 2.029–3.909). The association between tumor site (head, body, tail) and survival was no longer observed after adjustment for other prognostic factors.

## Discussion

11,672 new pancreatic cancer cases were identified among Shanghai residency during 2004 to 2009. The crude incidence rate of pancreatic cancer was increasing from 12.80/100,000 in 2004 to 15.66/100,000 in 2009, while the standardized incidence rate was about 6.70/100,000 and didn't change a lot in the same period. An overall 5-year survival rate 4.1% and a median survival of 3.9 months were computed in this large, population-based study. The survival rate and median survival time were greater in patients who were younger, diagnosed with localized disease, had well-differentiated tumors, had lower stage tumors and had received surgical resection than their counterparts. Surgical resection could prolong survival time after adjusted by demographic factors and tumor characters.

The incidence rate of pancreatic cancer was high and increasing from 2004 to 2009 among Shanghai residency, while the age-adjusted incidence rate was stable. So, the higher and increasing incidence rate might be attributed to the aging population in the advanced city like Shanghai. This incidence rate was similar to the China National Central Cancer Registry 2007 and 2009, with age adjusted incidence rate 7.28/100,000 in both 2007 [16] and 2009 [2]. The stable incidence rate of patients with pancreatic adenocarcinoma was also found in the Surveillance, Epidemiology and End Results program during 1977 to 2001, with incidence rate 11.3/100,000 in 1977–1981 and 10.9/100,000 in 1997–2001 [17]. Although different standard population was used, the incidence rate of pancreatic cancer among Shanghai residency was stable, just like those data in the whole country and United State.

The overall 5-year survival rate (4.1/100,000) and median survival time 3.9 months (95% CI 3.8–4.0) was equal to those discovered in western population-based studies as mentioned in the introduction section [4], [5], [6], [7]. For pancreatic cancer patients underwent surgical resection, we computed an overall 1-year survival rate (47.5%), 3-year survival rate (15.4%) and 5-year survival rate (10.1%). These survival rates were similar to those of 216 patients with pancreatic cancer treated in the first affiliated hospital of medical college of Zhejiang University between 1990 and 2000 (The 1-, 3- and 5-year survival rates in patients receiving radical resection were 44.71%, 14.98% and 9.99%, respectively) [18] and 2340 pancreatic cancer patients underwent radical resection in 14 hospitals in China between 1990 and 2000 (The 1-, 3- and 5-year survival rates were 54.36%, 13.47% and 8.47%, respectively) [9]. These survival rates were also comparable to Meyer's study on curatively resected ductal pancreatic adenocarcinoma who were operated on between 1986 and 1995 with 5-year survival rate 10.5% [19] and Distler's study on pancreatic adenocarcinoma underwent pancreatic head resection between 1993 and 2011 with 5-year survival rate 11.86% [20] and other hospital based studies in western countries [21], [22], [23]. The 5-year survival rates were higher than those in Huashan Hospital of Fudan University during 1970s (The 1-, 3- and 5-year survival rates were 50%, 25% and 0, respectively), while similar to those in 1980s (The 1-, 3- and 5-year survival rates were 57.1%, 28.5% and 9%, respectively) and 1990s (The 1-, 3- and 5-year survival rates were 61.1%, 27% and 11.1%, respectively) [24]. However, the survival rates were lower than some studies on pancreatic cancer patients underwent surgical resection in western countries [22], [25]. These differences and low survival rates may root in several reasons: 1) The most important reason may be the different time for diagnosis of pancreatic cancer and different type of cancer between China and western countries. Since there is no specific early symptom, pancreatic cancer is diagnosed only when the patients have symptoms of abdominal pain or jaundice. In the present study, 42.9% subjects were metastasis and only 7.5% subjects underwent surgical resection were well differentiated. It is usually too late for operation. 2) As mentioned by Gudjonsson [26], hospital-based follow-up studies tended to backtrack and figure out the actual number of survivors from the actuarial figures without knowing how many patients were lost to follow-up and when. Since Kaplan-Meier method has no limitation and no compulsory explanation on losses (censoring), the survival rates by Kaplan-Meier methods would be overestimated as increased censoring happened especially for those hospital-based pancreatic cancer studies. Survival estimates also may be biased (higher) if the censoring (i.e., loss to follow-up) is correlated with patients' death (e.g., poor survival and a large number of censored patients). Therefore, a possible explanation for our lower survival rate is that, unlike other studies, we used both active and passive follow-up of patients in our study. This allowed the actuarial survival rate computed by the Kaplan-Meier method in the present study to approach the actual survival.

Similar to previous studies [4], [5] [25], [27], this study found that younger age, localized disease, well-differentiated tumors, and surgical resection were related to better survival and longer median survival time. Because treatment is related to stage at diagnosis, we further evaluated treatment using multivariate Cox regression model to adjust demographic factors and tumor characteristics. Results showed that, regardless of age, stage at diagnosis and differential status, patients who had underwent surgical resection had better survival compared with those who received no surgical resection. This suggests that active initial treatment may prolong survival, even for patients with advanced disease. Although younger patients had better prognosis after surgical resection, some pancreatic patients older than 70 achieved over 5 years survival in the present study which indicated that elderly patients are able to withstand the stress of surgery and obtain the survival benefits achieved by younger patients.

This large, population-based study included all pancreatic cancer patients among Shanghai residency diagnosed from 2004 to 2009. Our use of active follow-up (contact of physicians' offices, hospitals, patients' relatives, and patients) in addition to passive follow-up including Shanghai vital statistics data allowed for a more complete vital status and survival assessment. Our use of active follow-up also allowed us to further evaluate long-term survivors and to confirm their pancreatic cancer diagnosis, which has been shown to be critical in small clinic-based studies [28].

Because our study used cancer registry data, detailed patient and tumor information that may influence treatment decisions were not available. With no information on surgical resection, we used pathology data to represent ‘surgical resection’. This may cause misclassification, especially for ‘non-surgical resection’ group, and lead to underestimation of difference between ‘surgical resection’ group and ‘non-surgical resection’ group. We also had no information regarding the use of specific surgical or chemotherapy or irradiation techniques. Despite these limitations, our study is the first population-based and most comprehensive recent analysis on survival rate and factors that influence the pancreatic cancer in mainland China. However, because of possessing the most advanced medical care and most experience experts on pancreatic cancer, our findings on the survival of surgical resection pancreatic cancer can't be regarded as a standard practice in China.

In summary, a lower overall survival rate was observed among patients diagnosed during 2004 to 2009 in our large, population-based study. Our results suggest that early detection and improved treatment strategies are needed to improve prognosis for this deadly disease.
