# Correction: The Incidence and Survival Rate of Population-Based Pancreatic Cancer Patients: Shanghai Cancer Registry 2004–2009

**DOI:** 10.1371/annotation/ca46077a-70c6-4ad2-9f40-565136259e5e

**Published:** 2014-01-07

**Authors:** Jianfeng Luo, Linhai Xiao, Chunxiao Wu, Ying Zheng, Naiqing Zhao

The first author, Jianfeng Luo, should be noted as contributing equally to this work along with the second and third authors, Linhai Xiao and Chunxiao Wu.

